# Effects of Kinesiotape versus Low-Dye Tape on Pain and Comfort Measures in Patients with Plantar Fasciitis: A Randomized Clinical Trial

**DOI:** 10.3390/life14020249

**Published:** 2024-02-12

**Authors:** Carmen García-Gomariz, David Hernández-Guillén, Pilar Nieto-Gil, Carlos Blasco-García, Montse Alcahuz-Griñán, José-María Blasco

**Affiliations:** 1 Department of Nursing, University of Valencia, Menéndez y Pelayo Av S/N, 46010 Valencia, Spain; carmen.garcia-gomariz@uv.es (C.G.-G.); pilar.nieto@uv.es (P.N.-G.); carlos.blasco@uv.es (C.B.-G.); montse.alcahuz@uv.es (M.A.-G.); 2 Group of Physiotherapy in the Ageing Process: Social and Health Care Strategies, Department of Physiotherapy, University of Valencia, Gascó Oliag 5, 46010 Valencia, Spain; jose.maria.blasco@uv.es; 3 Department of Physiotherapy, University of Valencia, Dr. Moliner 50 Av., 46100 Burjassot, Spain; 4 IRIMED Joint Research Unit (IIS-LaFe—UV), Dr. Moliner 50 Av., 46100 Burjassot, Spain

**Keywords:** plantar fasciitis, bandage, pain, randomized clinical trial

## Abstract

Background: Bandages are commonly used to relieve pain in patients with plantar fasciitis. The goal was to compare the effects of using kinesiotape versus low-dye tape in the acute phase of plantar fasciitis on pain and comfort measures. Methods: Forty individuals with plantar fasciitis were allocated to the kinesiotape or low-dye tape interventions. The patients were assessed at baseline and every 24 h until the fifth day. The primary measure was a visual analog scale of pain. The other measures were comfort, mobility, durability, personal hygiene, sweating, and allergies. The effects were compared with an ANOVA test, 95% CI. Results: Kinesiotape was more effective in reducing pain; the greater effect occurred during the first day, with a between-group difference of 2.0 (95% CI: 1.8 to 2.2). The pain differences between the treatments progressively reduced each day from the second day. Kinesiotape offered significantly higher performance than low-dye tape in mobility, comfort, and comfort in hygiene, sweating, and durability, with a large effect size *d* > 0.8. Conclusions: Kinesiotape could be more effective than low-dye tape in relieving pain in patients with plantar fasciitis, with a significant clinical impact on the first day of treatment. Kinesiotape can also provide higher performance in terms of comfort.

## 1. Introduction

Plantar fasciitis is a common musculoskeletal disorder characterized by pain involving the inferomedial aspect of the heel that is usually exacerbated following periods of non-weight-bearing when taking the first steps after waking up or when getting up after sitting [1,2]. It is the most common cause of musculoskeletal pain in the foot [3,4,5,6], has a prevalence between 8% and 25% in injured runners and athletes, and is usual in overweight people or those with lower physical function and quality of life [7,8,9]. The plantar fascia is a complex fibrous tissue that spans the entire foot. It begins at the medial calcaneal tubercle and connects to the phalanx, providing support to the arch and stability to the foot. This fascia bears weight during both stillness and movement, and it also serves as a spring that stores and releases elastic energy while walking. As we walk, the plantar fascia lengthens and shortens, allowing for efficient energy usage [10].

Patients with plantar fasciitis usually present with localized pain in the calcaneal tubercle during the first steps in the morning. The cause of plantar fasciitis is not completely known but is considered to be multifactorial. Factors such as foot weight-bearing activity at a higher frequency than usual, present with abnormal biomechanics, or delayed healing are likely contributors [11]. Overall, the etiopathogenesis evolves from an acute phase in which, over 4 weeks, the pain appears mainly when heel-supporting in the morning. This phase is followed by a subacute phase in which the heel pain increases both during dynamic movements and during prolonged standing [12]. After three months, it is considered chronic and a fasciopathy, with persistent and constant pain.

The diagnosis is usually clinical. Ultrasound observations can support the diagnosis when the thickening of the contralateral fascia is equal to or greater than 4 mm with a hypoechoic fascia [13]. The clinical course is not completely defined. However, one in ten cases requires surgery, while the remaining nine will be resolved with conservative treatment within 12 months [14]. Although different therapies can be applied to provide some level of pain relief, such as orthoses and splints, injections, extracorporeal shock wave therapy, platelet-rich plasma [15], or steroid injections [14], the outcome and the burden of the treatments vary markedly across protocols [16].

Among the possible conservative treatments, the use of bandages is a common practice, mainly in the acute phase. The bandage is usually used to treat pain, particularly as the palliative treatment in an early pathological phase but also as an adjunct or previous to other therapies. Traditionally, functional bandages with low-dye tape have been used with the idea of limiting the foot’s mobility and avoiding the biomechanical factors responsible for pain [17]. The therapeutic evidence of this solution has been classified at the medium level, surpassing anti-inflammatory or even manual therapy in effectiveness [12,18,19]. Intervention studies proved a short-term reduction of symptoms [15] but also some studies have compared the effectiveness of low-dye tape when combined with other therapies [6] or ultrasound therapy [20]. Among the advantages, low-dye tape can support the longitudinal arch but is probably primarily useful in the early stages of the condition and for pain management. A drawback is rigidity [21]. Problems with skin breakdown have also been reported [12]. Other drawbacks might include discomfort and restriction in mobility [22].

To avoid the above negative aspects, the so-called neuromuscular tape or kinesiotape could be a feasible alternative, with which the foot remains functional and there may be less sensation of tension. Among the roles attributed to kinesiotape are to provide support to injured muscles and joints, boost muscle strength, improve muscle function, decrease muscle tension, and alleviate pain by enhancing the skin and blood, as well as the lymph flow [23,24]. Additionally, it can help to reduce abnormal muscle tension, facilitate relaxation, and promote functional activities while assisting in the restoration of fascia and muscle function [25]. However, the literature supporting such a view in plantar fasciitis is scarce [18]; moreover, the effects of both low-dye tape and kinesiotape on important clinical aspects such as mobility, durability, comfort, hygiene, sweating, and allergy to the material are still understudied, and they have not been comprehensively compared [17,26,27,28].

Given the above factors, the goal of this randomized clinical trial was to compare the effects of using two types of bandages, i.e., kinesiotape versus low-dye tape, in the acute states of plantar fasciitis, on an essential aspect such as pain reduction but also on other aspects that are generally not considered in research and are important in clinical practice and to conduct daily activities with comfort, such as mobility, durability, comfort, personal hygiene, sweating, and allergies. This study hypothesized that kinesiotape can provide better performance and clinical outcomes than low-dye tape.

## 2. Materials and Methods

### 2.1. Design and Ethics

This study was designed as a two-arm randomized clinical trial involving patients diagnosed with plantar fasciitis, who were diagnosed using their symptoms—primarily pain—and complemented by an ultrasound test—a condition in case of a thickness of the contralateral fascia greater than or equal to 4 mm. Participants were divided into two groups for the study. The experimental group received an intervention with kinesiotape, while the control group received an intervention with low-dye tape. The study design adhered to the scientific and ethical guidelines set in Helsinki and was approved by the ethics and scientific board of the University of Valencia (no. 1940130). Participants were informed verbally and in writing about the trial and the implications of their participation, and all of them had to provide signed consent to participate. The study was supervised by the University of Valencia, and the procedures were conducted in the institution’s University Podiatric Clinic (CUP), between March and October 2022. The protocol was prospectively registered (Clinical Trials.gov id. NCT05384236).

### 2.2. Participants and Sample Size

The eligible sample were individuals over 18 years old who visited the facilities of the University Podiatry Clinic of the University of Valencia (Lluís Alcanys Foundation), in Valencia, Spain, to undergo a biomechanical exploration due to presenting clinical symptoms related to plantar fasciitis. In case of pain in the sole or heel, likely compatible with plantar fasciitis, the plantar fascia was assessed from its insertion in the calcaneus to the distal region, maintaining digital extension. Patients in the acute phase with signs consistent with plantar fasciitis were included if they presented (1) pain greater than 3 on a visual analog scale of pain (VAS-Pain), with 0 being the worst possible pain and 10 no pain, and (2) ultrasound observation indicating that the thickness of the affected plantar fascia was 4 mm greater compared to the contralateral unaffected foot [29]. The patients had to visit the clinic to be treated for the first time for a condition that had not recurred or been previously treated; if some potential participants previously underwent other therapeutic procedures, such as plantar supports or medication, they were excluded. The sample size required to provide adequate power to the trial was estimated before investigating by using the G*Power 3.19.4 program, and by setting an analysis of variance test with two groups, an expected minimal detectable 2-point score between-group difference on the visual analog scale of pain [30,31], and a confidence interval α set at 0.05; this analysis suggested that a total sample of 32 participants would provide power over 80%. Adding a likely sample loss of 20% and rounding to the nearest pair number, we estimated it necessary to recruit a total of 40 participants, who were divided into two equal groups.

### 2.3. Randomization and Masking

A clinician referred the potential participants who came to the facilities mentioned above for biomechanical exploration to the principal investigator, who conducted the clinical and echographic tests to make the diagnosis and verify compliance with the inclusion criteria. Participants who met the criteria, and who agreed and signed to participate, were sequentially and randomly assigned by the same researcher into the experimental or the control group, according to the output of a numerical sequence extracted with a random number generator (Matlab^®^, version: 9.13.0 (R2022b), Natick, MA, USA: The MathWorks Inc.; 2022) in origin. The principal investigator informed participants by telephone about the intervention date, without revealing the group assignment. However, participants were informed a priori about the possible treatments—before signing their consent; and there was the possibility that some participants, due to their experience or previous knowledge, were aware of the differences between the two bandages since they can be differentiated with the naked eye. Therefore, the blinding of participants was not assured. A podiatrist from the podiatry clinic of the University of Valencia with more than 10 years of experience was in charge of conducting both of the interventions. Therefore, the intervenor was not blinded. A member of the team, blinded to the interventions, was in charge of assessing participants and tabulating the data, which were anonymized with numerical codes in an Excel spreadsheet. Finally, another researcher used the coded database to conduct data analyses and extract results.

### 2.4. Interventions

**Figure 1:**
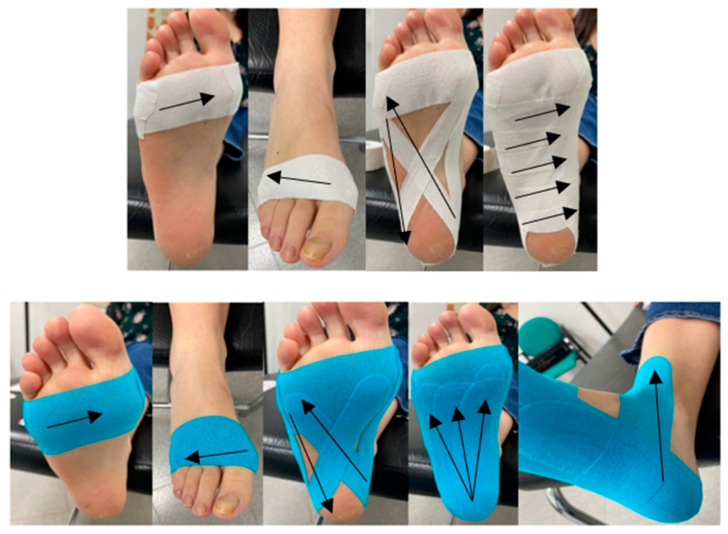
Low-dye tape (**up**) and kinesiotape (**down**) interventions. The arrows indicate the direction of the strips.

The experimental and control groups underwent the same intervention: the application of a bandage, but with different techniques and characteristics. The type of tape underpinned the difference between the experimental and control interventions: low-dye tape was applied to the 20 participants in the control group, while kinesiotape was applied to the 20 participants in the control group. Overall, the kinesiotape is more elastic, and the treatment was applied to increase the space in the area of pain and dorsal edema to reduce pain and relax the tension of the plantar fascia once the patient was recruited. The bandage used was a blue 5 × 5 cm Temtex-Kinesiology Tape^®^ (TEMTEX Towatek Korea Co., LTD; Gyeonggi-Do, Republic of Korea) [18]. The patient was first placed in a supine position on a stretcher. The footwear was removed, the foot was cleaned with a wet wipe and alcohol, and Tensospray^®^ (Willerby, UK) was applied along the entire path where the bandage was going to be placed to achieve a greater adherence. The ankle was held at 90 degrees and the leg was maintained in a neutral position. The technique, which is shown in Figure 1, is described in the following steps [18,32]: (1) placement of a strap in the area of the metatarsal heads at an approximate tension of 50%; (2) placement of a strap on the hind foot forming a circle with the front strap at 50% tension; (3) a figure eight was made on the sole, starting from the side of the fifth metatarsal, surrounding the heel and ending at the starting point. The tension of the strips was carried out just when the strips passed the point of pain at the insertion of the fascia in the calcaneus, approximately in its fourth quadrant, (4) the same as in step 3, but starting at the first metatarsal, so the tension of the strips was performed again just when the strips passed the point of pain at the insertion of the fascia in the calcaneus, approximately in its fourth quadrant; (5) placement of 3 active strips from the center of the heel, where the fascia insertion is located, toward the origin at the level of the metatarsals, the tension was approximately 60%; with nociceptive and space-releasing effects, (6) placing a strap at the main point of pain, that is, at the insertion toward the medial edge of the foot at a tension of 80 to 90% to reduce tension; and finally, (7) a band was placed at the level of the metatarsal heads as a 0% tension closure.

The participants in the control group underwent a similar treatment, but the technique was adapted to the application principles of low-dye tape in plantar fasciitis [27]. The tape was a Strappal^®^ (Willerby, UK) 3.7 × 10 cm in this case. This tape is more rigid than kinesiotape, and the technique was therefore different. Once the patient was recruited and informed and the consent was signed, the patient was placed supine on a stretcher, with the ankle at 90 degrees and the leg in a neutral position. Likewise, the footwear was removed, the area was cleaned with alcohol and Tensospray^®^ was applied along the entire route where the bandage was going to be placed for greater adherence. The technique is shown in Figure 1. No tension was applied due to the characteristics of the tape and it is described in the following steps. The first four steps were similar in terms of the path of the tape; however, no tension was applied: (1) placement of a strip in the area of the metatarsal heads, in the dorsal area (2) placement of a strap on the back, forming a circle with the front strap and in the plantar area; (3) a figure of eight was made, starting from the side of the fifth metatarsal, surrounding the heel and ending at the starting point, (4) the same operation was performed as in point (3) but starting from the hallux. The last part was different, as (5) a strip at the main point of pain, that is, at the insertion towards the medial edge of the foot, in the fourth quadrant of the calcaneus, as well as successive strips to make the closure up to the primary strip, under the metatarsal heads, with a total of 5 to 6 strips depending on foot length to relax the tension in the fascia [32].

### 2.5. Measures

Once the bandage was placed, the patients were informed about the procedure and were instructed that it was necessary to wear the bandage for a minimum of 4 to 5 days to complete the study. In addition, they were provided with assessment forms and instructed that each day they should write down the pain they felt, from the day before applying the bandage until its removal. The primary outcome was pain, measured with a visual analog scale of pain, in which 0 was no pain and 10 was the worst possible pain. The participants were requested to note their overall pain in the last 24 h to assess this outcome. Pain was assessed before the intervention at baseline, and then every 24 h until the fifth day of wearing the tape. Then, the pain was reassessed 24 h after removing the tape, i.e., pain before you put the bandage on, pain within 24 h of putting the bandage on, pain within 48 h of putting on the bandage, pain 3 days after putting on the bandage, pain 4 days after applying the bandage and pain 5–7 days after putting the bandage on (the day the bandage was removed). To assess this outcome, forms were provided to participants at baseline, and telephone calls were made to remind them to fill out the forms at the same time of day. Once the tape was removed, 0 to 10 visual analog scales were used to assess to assess the other aspects as secondary results. Specifically, these were the degree of foot mobility, comfort, and comfort in hygiene while wearing the tape. In addition, other factors were assessed to determine if the bandage had come off before the fifth day if sweating had been increased, and, if so, to what level, and if there had been any type of allergic reaction. Accordingly, the participants had to fill out and answer the following questions and scales. The degree of mobility of the foot when using the bandage had to be indicated on a scale in which mobility was graduated from 0 to 10; the degree of comfort of the bandage was graduated from 0 non-comfortable to 10 very comfortable; the degree of comfort in the hygiene of the bandage—for instance, when showering or personal hygiene—was 0 non-comfortable to 10 very comfortable; the participants were also asked whether the bandage had come off before meeting the 4 to 5 expected wearing days. If that was the case, they were instructed to indicate how many days the bandage was maintained, answering with yes or no and the number of days. They were also asked whether they had noticed a sweating increase when using the bandage, answering yes or no. If the answer to the previous question was positive, they had to indicate how much such sweating had increased using a 5-point Likert scale. Finally, the participants were asked whether they had any type of allergic reaction when using the bandage. The evaluation forms can be found in Appendix A. One week later, the participants were scheduled to attend the podiatry clinic to assess the progress of the treatment and hand out the questionnaires. The data were transferred to an anonymized database for analysis.

### 2.6. Data Analysis

A descriptive synthesis of the sample characteristics and an inferential analysis were conducted to evaluate the efficacy of the treatment, using the IBM SPSS Statistics 22.0 software, authorized by the University of Valencia. The sample distribution was tested with the Shapiro–Wilk test since the sample was below 50 individuals, and possible basal differences with *t*-tests or chi-square tests in case of continuous or qualitative variables, respectively. To test the hypothesis, the effects on pain were evaluated with an analysis of variance based on a generalized model of repeated measures, taking into account the factors of time (6 time points, i.e., baseline, 1, 2, 3, and 4 days of wearing the tape, and 1 day after removing it), group (experimental and control) and possible time per group interactions. The post hoc analysis was based on C-Dunnett and Tukey. The effect size according to Cohen’s *d* statistic was calculated and interpreted according to the limits for such a statistic, with *d* = 0.2, 0.5, and 0.8 considered as low, medium, or large effect sizes. A post hoc analysis was also conducted to determine the actual power of the trial. A one-way analysis of variance compared the between-group effects on the secondary outcomes. The confidence intervals were set at 95%.

## 3. Results

**Figure 2:**
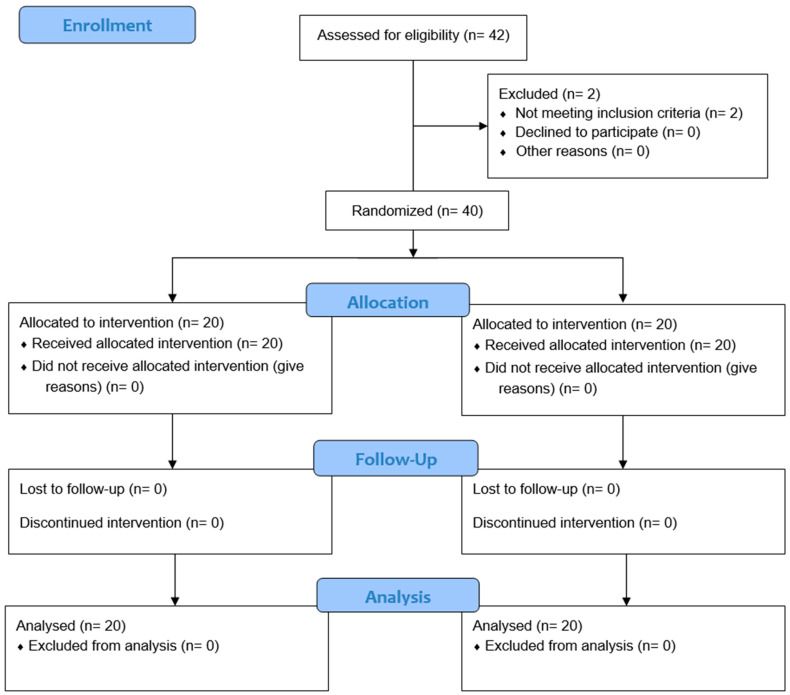
Flow chart of participants presented according to the CONSORT 2010 statement.

A total of forty-two individuals were carefully assessed, and among them, two participants had undergone drug treatment for pain or were already using plantar supports. Consequently, they were not included in the study. Finally, 40 individuals, aged 50 (23 to 66) years old, participated and were equally distributed into the groups. No participant presented with pathology in gastrocnemius, while the Foot Posture Index (FPI) indicated that no participant had a highly pronated or supinated foot. The sample was found to be normally distributed and indicated that there were no baseline differences observed between the groups. The absence of between-group basal differences suggested that the data were evenly distributed across the participants and that the randomization process was successful. The characteristics of the sample are shown in Table 1, while the flow of participants throughout the study is shown in Figure 2.

### Effects of the Intervention

**Figure 3:**
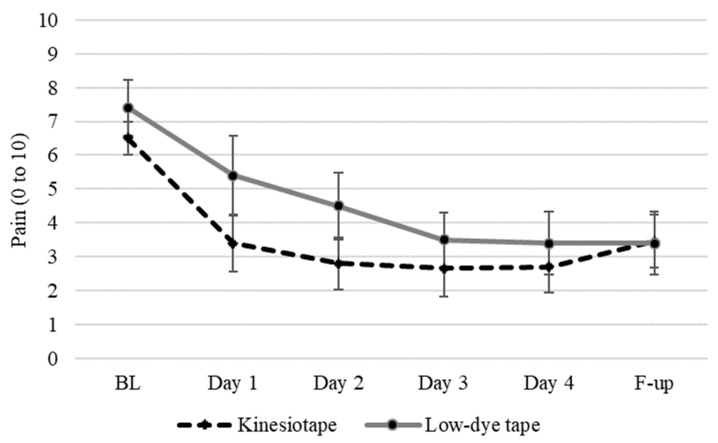
Average pain over the intervention in participants wearing kinesiotape or low-dye tape, from baseline assessment (BL) every 24 h until the fifth day, and then a 24 h follow-up evaluation once the tape was removed.

The pain was similar at baseline, with no between-group differences (*p* = 0.054). Then, there were time (*p* < 0.001), group (*p* = 0.022), and time per group differences (*p* = 0.002). Specifically, both treatments reduced the pain, but kinesiotape was more effective than low-dye tape in the first 24 h (2 points difference (95% CI: 1.8 to 2.2)) and 48 h (1.7 points difference (95% CI: (1.5 to 1.9)), with a large effect size and power over 80%. After that, both treatments evolved in an almost constant trend, with a slight increase in the average pain after removing the tapes, as shown in Table 2 and Figure 3.

Overall, the results also suggested that kinesiotape offered higher performances than low-dye tape in all the secondary measures, including mobility, comfort, comfort in hygiene, sweating, and a smaller number of times it was unstuck before the fifth day, suggesting that kinesiotape is also more effective in terms of comfort than low-dye tape. There were no allergic reactions, regardless of the tape.

Overall, most participants found kinesiotape highly comfortable, with 9 out of 10 being the most frequent score, while the distribution of answers with low-dye tape was very variable and ranged from 1 to 8. Similarly, participants referred to very good mobility with kinesiotape, mainly scored from 9 to 10, while participants using low-dye tape frequently scored mobility with 5 out of 10. The answer distribution in terms of comfort in hygiene was similar. Only participants using low-dye tape referred to sweating; this did not happen with kinesiotape. Finally, the durability was longer with kinesiotape. All the information is presented in Table 2, while Appendix B shows the distribution of the comfort answers.

The trial was adequately powered (>80%), with a large effect size for the pain measures assessed during the first 48 h. Then, the power reduced and there were no between-group significant differences. The trial was also adequately powered according to the comfort measures.

## 4. Discussion

This research was designed to assess, on the one hand, the effectiveness of kinesiotape compared to low-dye tape in terms of pain, and on the other hand, to evaluate additional factors that are generally not studied exhaustively but which are also important and must be taken into account when healthcare professionals choose to use one or another treatment, such as comfort, better mobility, sweating or hygiene. The results of this research support two major findings. First, therapy with low-dye tape and kinesiotape appeared to be effective in reducing pain in the acute phase of plantar fasciitis. However, the therapy with kinesiotape proved to be more effective in reducing pain than with low-dye tape during the first 48 h (with a two-point clinically important difference in the first 24 h), whereas the therapeutic effect was no longer present when the strips were removed from both bandages. The second finding suggested that the kinesiotape offered better performance than the low-dye tape in all the secondary measures; specifically, in terms of improved mobility, comfort, comfort in hygiene, reduced sweating, and loss of adherence to the skin. These findings support that treatment with kinesiotape in people with plantar fasciitis in the acute phase is more effective in reducing pain, and overall, more comfortable, than with low-dye tape.

As for pain, the main finding was that participants using kinesiotape presented a higher and statistically significant pain reduction compared to those using low-dye tape at most time points. In addition, the results allowed a more specific interpretation of such a statement in terms of the effects of each type of tape and the durability of the effects. Although kinesiotape was more effective, the fact was that low-dye tape was also effective, since there was a two-point detectable difference after 1 day of treatment [29,30], and the pain continued to reduce until the third day. From then on, the pain remained almost constant, and there was no evolution in the following assessments. A similar trend could be observed in the participants using kinesiotape, but the pain reduction was higher in this case, which allowed us to state the main finding above. The greater between-group differences were present during the first two days of treatment, with a two-point clinically important difference on the first day. Still, although statistically significant, the pain differences were reduced from the third day onwards. This suggests the possible clinical application regarding the use of tapes since maximum effectiveness will be achieved during the first days. Future studies could be aimed at determining whether changing the bandage for a new one would be appropriate after this time, or even if applying new bandages could achieve greater improvements in terms of pain.

At present, we have not found research that directly compared the effects on pain of low-dye tape and kinesiotape in patients with plantar fasciitis, so it has not been possible to make direct comparisons with previous studies. The short-term benefit in the treatment of plantar fasciitis through taping techniques had been studied, but not assessing either low-dye tape [33], kinesiotape [34], or even other types of solutions, such as the calcaneal bandage [17] or the so-called quick tap [35]. It is possible to find in the literature cases described in which different types of bandages were used, including low-dye tape, and in which no significant difference was found between one technique and another. We have only found one study that compared the two bandages, but it was carried out from a biomechanical point of view, not pain, since they focused on aspects such as possible differences in the maximal subtalar eversion, the navicular height or maximal strains on the fascia band among others [36]. Therefore, to the best of our knowledge, this is the first clinical trial in which the two techniques have been comprehensively compared in individuals with plantar fasciitis.

Having said that, some studies focused on other pathologies and conditions in which these bandages were used with other adjuvant techniques, such as the placement of plantar support with reinforcement of the internal longitudinal arch [4], the application of ultrasound [6] of shock waves [37] or stretching of the posterior musculature of the lower extremities [5]. Finally, Bahar-Ozdemir and Atan [37] introduced kinesiotape using the low-dye tape technique under the name kinesio low-dye tape and compared it with shock waves and a placebo. In some cases, the available studies had a high risk of bias or low methodological quality [17]. Our comparison in this study contributes to the literature demonstrating which treatment may be more effective, and hence its clinical importance, not only at the level of pain but also at the level of comfort.

The main therapeutic objective of plantar fasciitis is to reduce pain. However, secondary aspects, such as comfort, sweating or allergies, and durability, are desirable factors that must be taken into account when applying one or the other option. In this regard, kinesiotape presented higher performance than low-dye tape. The histograms in Appendix B support such a statement. First, kinesiotape was highly comfortable against low-dye tape and mostly scored 9 out of 10 in these terms. It presented more comfort in hygiene since most of the answers were scored over 6 out of 10; by contrast, participants using low-dye tape referred to the opposite. Importantly, no participant referred to sweating with kinesiotape, but some of those who used low-dye tape did. Finally, most kinesiotape lasted until the end of the treatment without being unstuck; however, the low-dye tape of approximately half of the control sample was unstuck from days 2 to 4. Overall, this study found that kinesiotape was significantly more effective than low-dye tape in all the comfort measures.

Some previous studies had assessed these aspects; however, they did so in populations and/or conditions other than the ones proposed in this work. For instance, durability and comfort in the iliotibial band syndrome, with the question “Do you think kinesio tape is comfortable?”, although it was in healthy athletes [38], or the use of kinesiotape to assess comfort in breast cancer lymphoedema [39], or in the patellar tendon, a study in which the authors assessed the comfort and ground reaction forces in flat-footed female runners and used a 150 mm visual analog scale [40], among others such as durability [41]. In these terms, our study revealed that kinesiotape may be the best of the studied bandage options if we attend to aspects related to comfort and durability. None of these factors had been evaluated with low-dye tape.

An important limitation to be noted is that therapy with tape is not considered a treatment that resolves plantar fasciitis by itself, and clinicians usually combine this solution with other options, as supported in the literature. For instance, the placement of a plantar support with reinforcement of the internal longitudinal arch [4], the application of ultrasound [6], shock waves [37], or the stretching of the musculature of the lower extremities [5], among others. Yet, one strength of this study has been to demonstrate its usefulness and efficacy in reducing pain in an acute phase, regardless of whether or not the tape is combined with another therapy. This is a finding that can be taken into consideration in clinical practice, especially in those cases in which the definitive treatment is being designed, manufactured, or awaiting completion, e.g., in the manufacturing process of plantar supports, since it is possible to significantly alleviate the acute pain and even avoid taking pharmacological treatment using tape.

In addition to the limitations previously discussed, we need to note that there are no studies with which to compare our results directly. Randomization was successful, with no between-group baseline pain differences. However, the *p*-value was 0.054 and should be taken into account when interpreting the results. It should be remembered that although kinesiotape could reduce pain more than a common tape, its application is usually planned in an acute phase and always as a complementary therapy, which should be considered both in clinical practice and future studies, insisting on the clinical importance provided by our results when selecting the appropriate bandage, not only for the improvement in pain reduction but also for convenience and comfort, and according to our findings, kinesiotape should be the bandage chosen in the acute phase of plantar fasciitis. Finally, we believe that the therapeutic combination with plantar supports that aims to reduce the tension to which the plantar fascia that has caused fasciitis may be subjected is of special importance, which serves as the design for future research.

## 5. Conclusions

This study supports that kinesiotape is more effective than low-dye tape in relieving pain in patients in the acute phase of plantar fasciitis. In addition, kinesiotape offered better benefits in mobility, comfort, comfort in hygiene, sweating, and durability. The greater pain difference was present during the first day of wearing the tapes, but such a difference was reduced from the second day onwards. Future studies could be aimed at reliably demonstrating both the optimal time for which a bandage should be worn based on its effectiveness in relieving pain, and the effect that changing the bandages would have after wearing them for two or three days, that is, the time at which our study suggests that efficacy is reduced. Overall, the findings should be taken into account by healthcare professionals in terms of the treatment of choice regarding the use of bandages aimed at alleviating the pain of patients with plantar fasciitis.

## Figures and Tables

**Table 1:** Baseline characteristics of the sample.

	Kinesiotape (*n* = 20)	Low-Dye Tape (*n* = 20)	*p*-Value
Sex (men/women)	6/14	7/13	0.736
Age (years)	52.5 (46.2 to 58.8)	47.6 (43.4 to 51.8)	0.217
BMI (kg/m^2^)	25.3 (23.6 to 23.9)	27.5 (24.9 to 30)	0.163
Foot size (EU)	39.8 (38.8 to 40.7)	40.2 (38.9 to 41.4)	0.580
VAS pain (0–10)	6.5 (5.7 to 7.2)	7.4 (7.3 to 7.5)	0.054

Data are given as the mean (95% CI). *p*-value for a *t*-test or chi-square test in case of continuous or categorical variables, respectively. BMI = Muscle Mass Index; EU = European Union; VAS = Visual Analogue Scale.

**Table 2:** Effects of the interventions.

	Time Point	Kinesiotape (n = 20)	Low-Dye Tape (n = 20)	Group Difference	*p*-Value (Time)	*p*-Value (Group)	Cohen *d*
Pain (0 to 10)	1st day	3.4 (2.4 to 4.4)	5.4 (4.6 to 6.1)	2.0 (1.8 to 2.2)	2–1, 2–3, 2–4, 2–5, 2–6	0.023	0.9 (0.3 to 1.6)
	2nd day	2.8 (1.9 to 3.6)	4.5 (3.8 to 5.1)	1.7 (1.5 to 1.9)	3–1, 3–2, 3–3, 3–4, 3–5	0.002	0.9 (0.3 to 1.6)
	3rd day	2.7 (2 to 3.4)	3.5 (2.8 to 4.2)	0.9 (0.7 to 1.1)	4–1, 4–2, 4–3	0.054	0.6 (0 to 1.1)
	4th day	2.7 (1.9 to 3.5)	3.4 (2.7 to 4.1)	0.7 (0.5 to 0.9)	5–1, 5–2, 5–3, 5–5	0.100	0.4 (−0.2 to 1)
	F-up	3.5 (2.7 to 4.3)	3.4 (2.7 to 4.1)	0.1 (−0.1 to 0.2)	6–1, 6–2	0.464	0 (−0.5 to 0.5)
Mobility (0 to 10)	F-up	9.3 (8.8 to 9.7)	5.8 (5 to 6.6)	3.5 (3.3 to 3.7)	-	<0.001	2 (1.4 to 3)
Comfort (0 to 10)	F-up	9.0 (8.6 to 9.3)	5.3 (4.4 to 6.1)	3.7 (3.5 to 3.9)	-	<0001	2.4 (1.5 to 3.1)
Comfort in hygiene (0 to 10)	F-up	7.4 (6.8 to 8.0)	3.8 (3.2 to 4.4)	3.6 (3.4 to 3.7)	-	<0.001	2.5 (1.7 to 3.4)
Taken off (n, %)	F-up	2 (10%)	9 (45%)	7 (35%)	-	0.013	
Sweating (yes, %)	F-up	0 (0%)	11 (55%)	11 (55%)	-	<0.001	
Sweating (0 to 10)	F-up	0 (0)	2.7 (2.4 to 2.9)	2.7 (2.5 to 2.9)	-	<0.001	7.6 (5.8 to 9.4)

Data are given as the mean (95% deviation confidence interval). F-up = 24 h follow-up after removing the tape. *p*-values of the analysis of variance of repeated measures to assess time (*p* < 0.05) and group differences in the case of pain. *p*-values to assess group differences with unpaired *t*- (continuous) and chi-square (categorical) tests in the case of secondary measures. *p*-value (time) referred to differences in the times 1 (basal) to 6 (follow-up) assessments.

**Figure A1:**
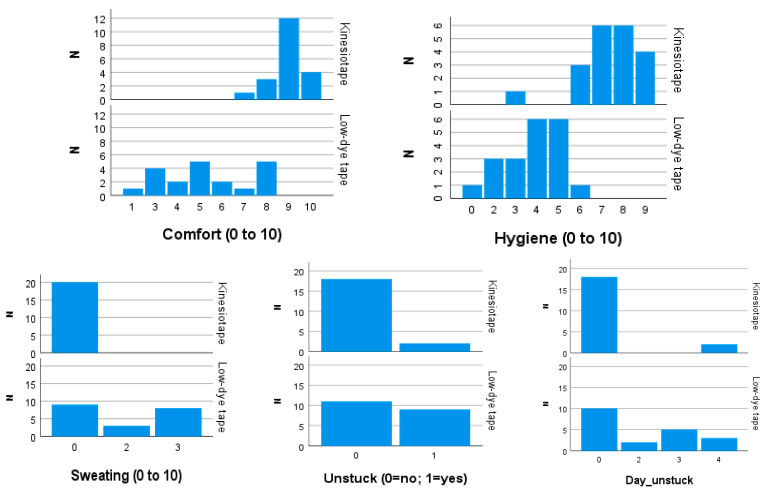
Distribution of answers to the comfort measures.

## Data Availability

The raw data supporting que conclusions of this article will be made available by the authors on request.

## Appendix A. Evaluation Form

**Table A1:** Evaluation form.

Pain before you put the bandage on													
No pain	0	1	2	3	4	5	6	7	8	9	10	The worst pain	
Pain within 24 h of putting the bandage on													
No pain	0	1	2	3	4	5	6	7	8	9	10	The worst pain	
Pain within 48 h of putting on the bandage													
No pain	0	1	2	3	4	5	6	7	8	9	10	The worst pain	
Pain 3 days after putting on the bandage													
No pain	0	1	2	3	4	5	6	7	8	9	10	The worst pain	
Pain 4 days after applying the bandage													
No pain	0	1	2	3	4	5	6	7	8	9	10	The worst pain	
Pain 5–7 days after putting the bandage on (the day you remove the bandage)													
No pain	0	1	2	3	4	5	6	7	8	9	10	The worst pain	
Assess the degree of mobility of the foot when using the bandage													
Non-mobility	0	1	2	3	4	5	6	7	8	9	10	High mobility	
Assess the degree of comfort of the bandage													
Non-comfortable	0	1	2	3	4	5	6	7	8	9	10	Very comfortable	
Assess the degree of comfort in the hygiene of the bandage (for example when showering or personal hygiene)													
Non-comfortable	0	1	2	3	4	5	6	7	8	9	10	Very comfortable	
Has the bandage come off before you meet all the days listed? If yes, please indicate how many days the bandage has been maintained													
Yes			No				Days:						
Has sweating increased when using the bandage									Yes			No	
If you answered yes to the previous question, indicate how much your sweating has increased													
Very little	1		2		3		4		5		A lot		
Have you had any type of allergic reaction when using the bandage?									Yes			No	

## Appendix B. Distribution of Answers to the Comfort Measures

There were no allergies, so the graph has not been represented.

The figures show the distribution of the answers as histograms, where the y-label N is the number of participants and the x-label is the measure of comfort, comfort in hygiene, sweating, and durability, as measured in terms of whether the bandage was unstuck before completing the intervention.
